# Effect of Mo_2_C Addition on Microstructure and Wear Behavior of HVOF Carbide-Metal Composite Coatings

**DOI:** 10.3390/ma18245622

**Published:** 2025-12-15

**Authors:** Feichi Chen, Xiang Xia, Wei Wang, Xiufang Gong, Xiaohu Yuan, Chunmei Tang, Xia Lou, Zhixing Guo, Longgang Wang, Bin Wu, Yunyi Zhu, Mei Yang

**Affiliations:** 1 College of Materials and Chemistry & Chemical Engineering, Chengdu University of Technology, Chengdu 610059, China; fz17711560jia@163.com (F.C.);; 2 School of Mechanical Engineering, Sichuan University, Chengdu 610065, China; 13980534090@163.com (X.X.);; 3 State Key Laboratory of Clean and Efficient Turbomachinery Power Equipment, Deyang 618000, China; 4 Dongfang Electric Corporation Dongfang Turbine Co., Ltd., Deyang 618000, China

**Keywords:** HVOF, WC-10Co4Cr coating, Mo_2_C, wear resistance, wear mechanism

## Abstract

In this study, carbide-metal composite coatings (WC-10Co4Cr) were prepared via high-velocity oxygen-fuel (HVOF) spraying, and the influence of Mo_2_C addition on the microstructure, mechanical properties, and wear performance was systematically investigated. The results indicate that Mo_2_C is solid-soluted in WC during the preparation process, which induces lattice distortion. Mo_2_C addition results in refinement of the grain size of WC particles, homogenization of the binder phase distribution, and reduction of the porosity of the coatings. An appropriate amount of Mo_2_C addition significantly enhances coating performance. The coating containing 2 wt.% Mo_2_C exhibited optimal properties. It demonstrated the highest microhardness and the lowest porosity, and wear tests revealed it had the lowest friction coefficient and wear rate at room temperature, which is primarily due to enhanced hardness and density that effectively suppressed abrasive wear. At 400 °C, the coating with 2 wt.% Mo_2_C addition also showed the most stable and lowest friction coefficient. The generated Mo-containing oxides acts as a solid lubricant, isolating friction surfaces and mitigating both oxidative and adhesive wear. However, excessive Mo_2_C content leads to an abnormal increase in the volume fraction of the binder phase, accompanied by reduced hardness. This induces a transition of the wear mechanism toward adhesive wear dominance, with complex nonlinear evolution characteristics.

## 1. Introduction

Surface wear and corrosion are primary causes of mechanical component failure, making the enhancement of material surface properties crucial for extending service life [1]. Critical components in high-parameter steam turbines and heavy-duty gas turbines-including turbine blades, impellers, valves, burner attachments, and sliders-are simultaneously exposed to oxidation, erosion, and wear under high-temperature conditions. These extremely severe operating environments impose stringent requirements on the comprehensive performance of protective coatings, which require a low friction coefficient, excellent high-temperature stability, wear resistance, and corrosion resistance.

Currently, metal carbides are extensively utilized across various fields, including electronics [2], nanotechnology [3], and nuclear technology [4]. Among them, carbon-metal composite coatings have been widely applied for the protection of high-parameter gas turbines and steam turbines. These coatings utilize carbide particles as the reinforcing phase, providing high hardness and outstanding wear resistance, along with significantly better high-temperature oxidation resistance compared to single-metal coatings [5,6,7]. Among them, the WC-10Co4Cr coating is extensively applied for wear protection under various harsh service conditions due to its good corrosion resistance, high-temperature resistance, and abrasion performance. Common techniques for preparing WC-10Co4Cr coatings include HVOF [8], HVAF [9], APS [10], and laser cladding [11]. Among them, high-velocity oxygen-fuel (HVOF) spraying has shown significant potential in the fabrication of cermet coatings due to its relatively low processing temperature, high coating bond strength, and dense microstructure [12,13]. However, with the advancement of steam and gas turbine technology toward higher operational parameters, increased stress levels, and extended service life, the performance of conventional WC-10Co4Cr coatings under ultra-high temperatures, high stress, and long-term service conditions has gradually become inadequate to meet practical requirements.

Studies have shown that the performance of WC-10Co4Cr coatings can be enhanced through compositional optimization, such as the addition of oxides and carbides into the WC-based coatings. Shi et al. [14] fabricated WC-10Co4Cr coatings with Al_2_O_3_ addition and found that the addition of Al_2_O_3_ reduced the coefficient of friction (COF) and improved the wear resistance of the coatings. Xi et al. [15] fabricated WC-10Co4Cr-xTiC coatings via laser cladding and investigated the effect of TiC on their tribological properties. The results demonstrated that TiC addition contributed to reduced porosity and enhanced wear resistance. Studies indicate that the addition of Mo element to the alloy can improve the wettability between the binder phase and the hard phase, thereby promoting coating density, reducing defects, and enhancing both the density and mechanical properties of the coating [16,17]. Cui et al. [18] fabricated CoCrNi, CoCrW, and CoCrMo alloys using powder metallurgy and systematically compared their tribological behaviors at elevated temperatures. The results indicated that the Mo-enhanced CoCr-based alloy exhibited the highest hardness and the best high-temperature tribological performance. Netrananda et al. [19] investigated the effect of Mo on the microstructure and high-temperature dry sliding wear behavior of WC-based coatings. They prepared a WC-CoCr/10%Mo coating via HVOF and found that the formation of MoO_3_ during high-temperature wear helped reduce both the wear rate and the friction coefficient, further confirming the beneficial role of Mo. Guo et al. [20] prepared Mo_2_C-doped WC-TiC-Ni cemented carbides via vacuum sintering. The addition of Mo_2_C resulted in refined WC grains, enhanced hardness, and improved transverse rupture strength. Although these studies demonstrate the potential of Mo in enhancing the tribological properties of coatings, systematic research on the influence of Mo_2_C content on the performance of WC-10Co4Cr coatings remains scarce. In particular, the effects of different Mo_2_C mass fractions on the mechanical properties, microstructural evolution, and wear mechanisms of the coatings are still not well understood.

Therefore, this study aims to address this gap. WC-10Co4Cr powders with different amounts of Mo_2_C added were prepared using the agglomeration-sintering process. Corresponding coatings were deposited on substrates via HVOF. The influence of Mo_2_C content on the microstructure, mechanical properties, friction coefficient, and wear rate of the coatings was systematically analyzed, and the underlying wear mechanisms were thoroughly investigated. The findings are expected to provide a theoretical basis and data support for the compositional design and engineering application of high-performance WC-based coatings.

## 2. Experimental

### 2.1. Preparation of Mo_2_C-Containing Feedstock Powders

**Figure 1:**
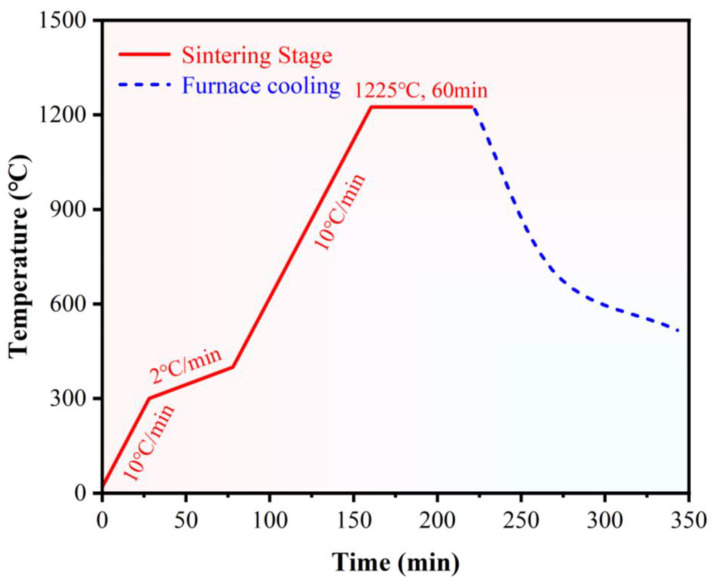
Sintering temperature curve.

High-purity powders of WC, Co, Cr, and Mo_2_C were used as the starting materials in this study. Powders of WC-10Co4Cr with Mo_2_C mass fractions of 0%, 2%, 4%, and 6% were designed and prepared. The nominal composition of the Feedstock powder is shown in Table 1. According to the design, the raw powders were weighed and loaded into a planetary ball mill together with alcohol. The ball-to-powder mass ratio was set at 3:1. The mixture was milled and blended at a speed of 350 rpm for 4 h. After ball milling, 5 wt.% PEG4000 was added as the forming agent. After milling and sieving, the thoroughly mixed slurry was pumped into a powder spray dryer for spray granulation, with an inlet air temperature of 220 °C, an outlet air temperature of 95~100 °C, and an atomizer frequency of 225 Hz. The powder was then sintered in a vacuum sintering furnace at 1225 °C for 1 h. The sintering temperature curve is shown in Figure 1. The powder was sieved to obtain a particle size range of 15~45 μm for use as the feedstock for HVOF spraying.

### 2.2. HVOF Sprayed Coating

The coatings were fabricated using the as-prepared powders by high-velocity oxygen-fuel (HVOF) spraying technology. The spraying parameters were set as follows: oxygen flow rate of 920 SL/min, argon flow rate of 3.7 SL/min, kerosene flow rate of 22.7 L/h, spray distance of 350 mm, and chamber pressure of 0.75 MPa. The substrate material was AISI 316L stainless steel. To ensure good bonding between the coating and the substrate, the substrate surface was pre-treated with grit blasting before spraying to remove contaminants and increase surface roughness.

### 2.3. Characterization

Phase analysis of the samples was performed using X-ray diffraction (XRD, PW-1700, Philips, Amsterdam, The Netherlands) with Cu Kα radiation. The scanning range was from 10° to 90° at a scan rate of 0.026°∙s^−1^. The microstructure of the thermal spray powders, as well as the surface, cross-section, and wear tracks of the coatings, was analyzed using scanning electron microscopy (SEM, S-4800, Hitachi Co., Tokyo, Japan). Energy-dispersive X-ray spectrometry (EDS) was employed for elemental composition analysis. The porosity within the coating cross-section was measured using Image-J software (version 1.54p). It was expressed as the percentage of pore area to the total cross-sectional area. The measurements were conducted on five randomly selected regions at a magnification of 500×, and the mean value was reported as the experimental result. The microhardness of the coatings was measured using an HVD-1000 digital display microhardness tester (Shanghai Jujing Precision Instrument Manufacturing Co., Ltd., Shanghai, China) with a test load of 2.94 N and a dwell time of 15 s. Ten indentations were made on each coating. Wear resistance was characterized using an HT-1000 friction (Lanzhou Zhongke Kaihua Technology Development Co., Ltd., Lanzhou, Gansu, China) and wear tester. Under standard atmospheric pressure, tribological tests were conducted at room temperature and 400 °C for 20 min, using a friction pair consisting of the sample and a ZrO_2_ ball with a diameter of 6 mm. The test parameters were set to a rotational speed of 200 rpm, a normal load of 10 N, and a wear track radius of 2 mm. For the 400 °C tests, the sample was first heated to the target temperature and maintained under isothermal conditions for 20 min to ensure temperature uniformity before initiating the wear test. Three coated samples were tested for each coating. The total volume loss of the coated specimens during testing was estimated using a 3D profilometer (Bruker Corporation, Billerica, MA, USA).

**Figure 2:**
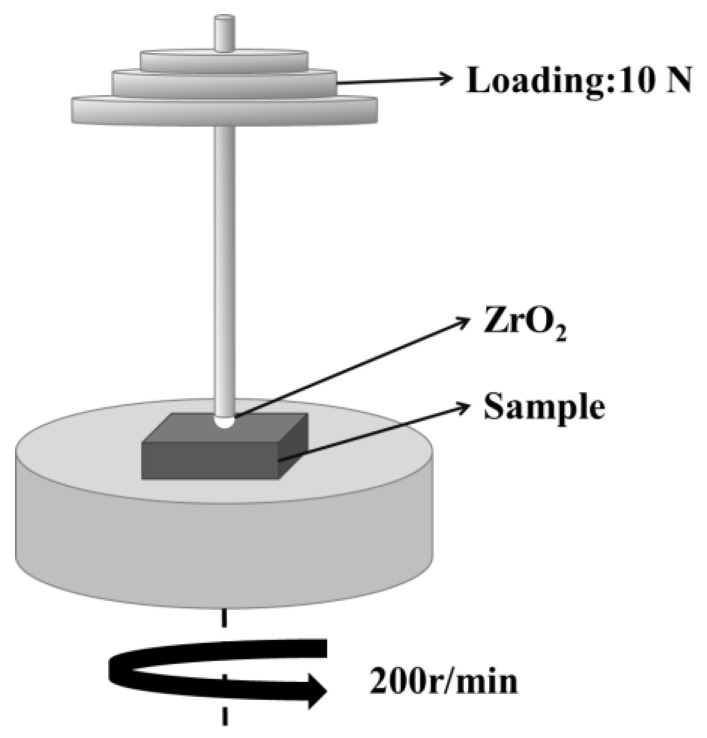
Diagram of the friction test.

The volume loss of all coating specimens was converted to a specific wear rate, defined as K_S_ = V/(P∙S), where V is the volume loss of the specimen (mm^3^), P is the normal load (N), and S is the sliding distance (m). The schematic diagram of the friction testing machine is revealed in Figure 2.

## 3. Results and Discussion

### 3.1. Characterization of Feedstock Powder

**Figure 3:**
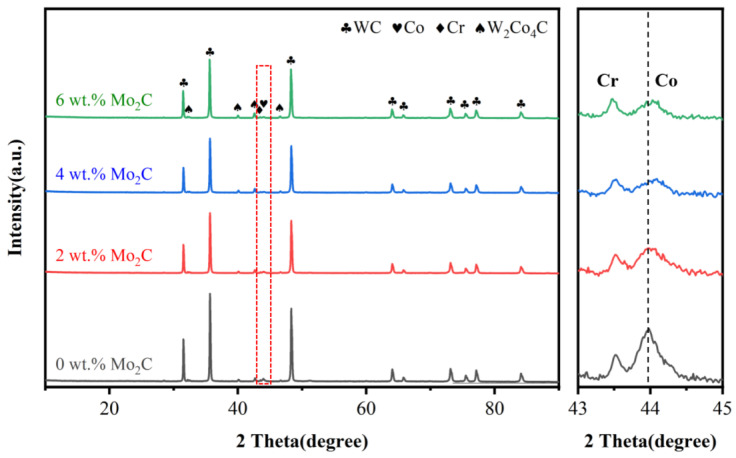
XRD patterns of WC-10Co4Cr feedstock powders with different Mo_2_C additions.

Figure 3 shows the XRD patterns of WC-10Co4Cr feedstock powders with different Mo_2_C additions. The powders are predominantly composed of the WC phase, alongside minor Co, Cr, and W_2_Co_4_C phases. The weak diffraction peaks of Co and Cr are attributed to their low content and solid solution effects. With the addition of Mo_2_C, no distinct Mo_2_C diffraction peaks are detected, and the intensity of the WC peaks decreases. This could be due to the dissolution of Mo_2_C into the WC lattice, forming a (W,Mo)C solid solution [21]. Figure 3 shows a magnified XRD pattern in the 2θ range of 43° to 45°. After Mo_2_C addition, the intensity of the Co diffraction peak weakens, and the peak position shifts to higher angles, which may be attributed to the formation of intermetallic compounds between Mo and Co/Cr [22].

**Figure 4:**
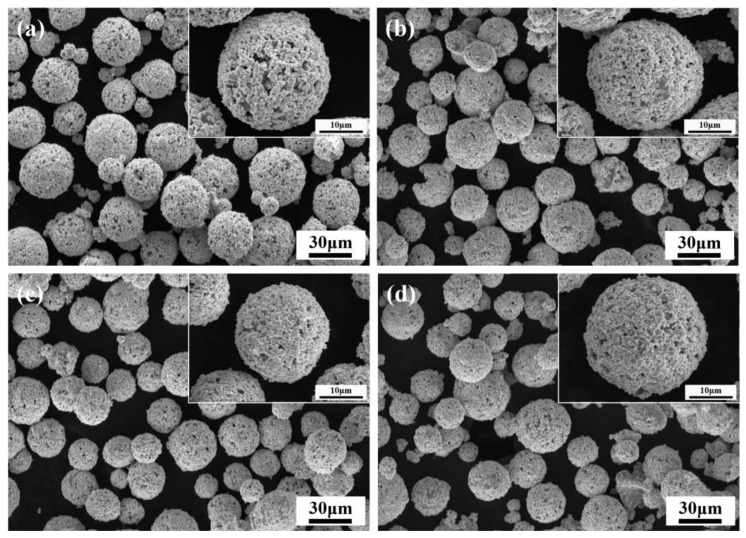
SEM micrographs of WC-10Co4Cr feedstock powders with different Mo_2_C additions: (**a**) 0 wt.% Mo_2_C; (**b**) 2 wt.% Mo_2_C; (**c**) 4 wt.% Mo_2_C; (**d**) 6 wt.% Mo_2_C.

**Figure 5:**
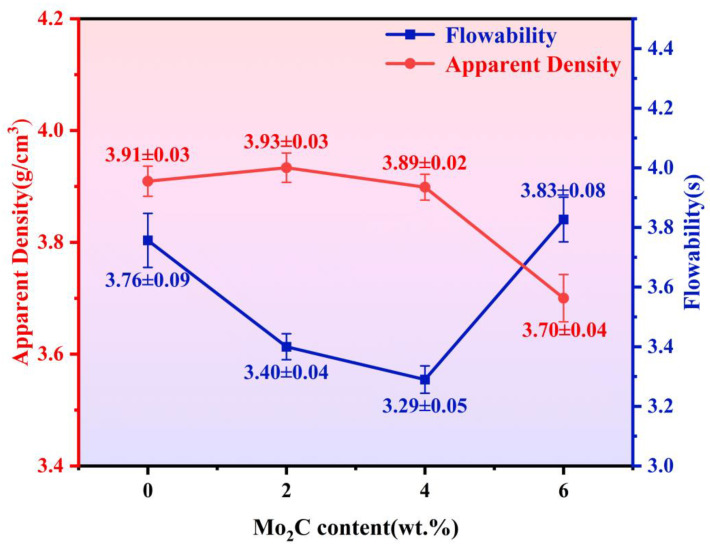
Flowability and apparent density of the feedstock powders.

Figure 4 presents the SEM micrographs of WC-10Co4Cr feedstock powders with different Mo_2_C additions. The powders primarily consists of intact spherical particles with high sphericity, demonstrating minimal fracture or agglomeration. It exhibits a uniform particle size distribution ranging from 10 to 45 μm, with most particles measuring approximately 30 μm. Powders sphericity significantly determines particle trajectories in the flame stream. Regular-shaped particles maintain stable flight paths, enabling the formation of uniform splats upon substrate impact and dense packing with low porosity. A consistent particle size distribution ensures similar flight trajectories among different particles, contributing to relatively uniform and dense coatings. With Mo_2_C addition, powders surface porosity decreases and its distribution becomes more homogeneous. As shown in Figure 5, the addition of Mo_2_C initially enhances then reduces powder flowability, with optimal flow achieved at 4 wt.% Mo_2_C. When the Mo_2_C content increased from 0% to 4%, the apparent density of the powder varied only slightly, and the differences fell within the margin of error. However, when the Mo_2_C content reached 6%, the apparent density showed a decreasing trend.

### 3.2. Microstructures and Properties of HVOF Coating

**Figure 6:**
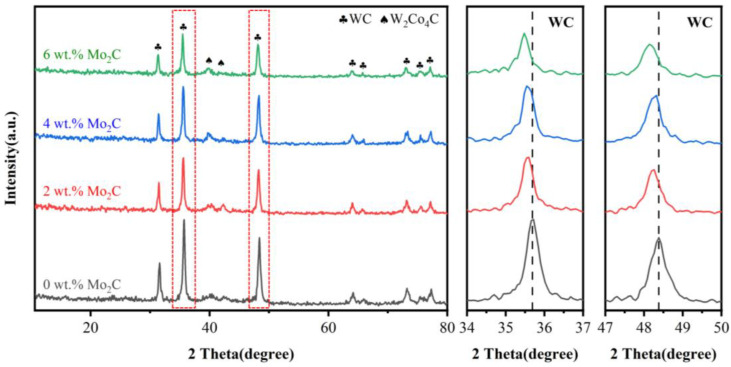
XRD patterns of WC-10Co4Cr coatings with different Mo_2_C additions.

Figure 6 presents the XRD patterns of WC-10Co4Cr coatings with different Mo_2_C additions. The coatings are primarily composed of WC phase with minor W_2_Co_4_C phase. The magnified patterns in the 2θ ranges of 34° to 37° and 47° to 50° reveal that the WC diffraction peaks shift toward lower angles with Mo_2_C addition, resulting from the dissolution of hexagonal Mo_2_C into the hexagonal WC lattice to form (W,Mo)C solid solution. Compared with the feedstock powders, the diffraction peaks of Co and Cr binder phases become significantly weaker in the coatings. This can be attributed to the low content of Co and Cr.

**Figure 7:**
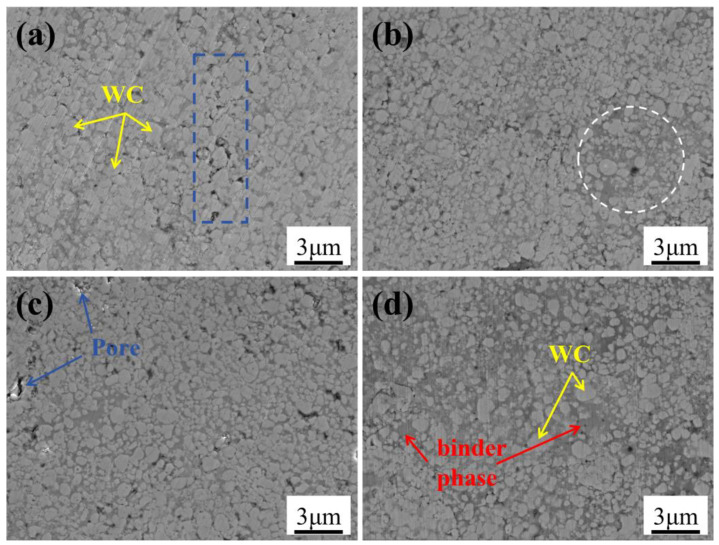
SEM micrographs of WC-10Co4Cr coatings with different Mo_2_C additions: (**a**) 0 wt.% Mo_2_C; (**b**) 2 wt.% Mo_2_C; (**c**) 4 wt.% Mo_2_C; (**d**) 6 wt.% Mo_2_C.

Figure 7 presents the SEM micrographs of WC-10Co4Cr coatings with different Mo_2_C additions. The dark gray regions correspond to the CoCr binder phase, while the bright white areas represent WC particles distributed within the binder matrix that fills interparticle voids. The area fraction of the binder phase in different coatings was calculated using Image-J software based on SEM images. When no Mo_2_C was added, the area fraction was 22.26%; at a Mo_2_C content of 2 wt.%, it was 24.71%; at 4 wt.%, it was 30.88%; and at 6 wt.%, it reached 42.09%. As shown in Figure 7a, the coating without Mo_2_C addition displays predominantly irregular polyhedral WC grains ranging from 0.1 μm to 1.47 μm in size. As evidenced in the regions marked by blue dashed lines, inadequate penetration of the CoCr binder between hard phase particles results in residual pores. With 2 wt.% Mo_2_C addition, the grain size decreases to 0.1 μm–1.34 μm. Selected areas exhibiting abnormal increases in binder phase volume fraction are marked with white dashed lines in Figure 7b, while the coating surface shows a concurrent reduction in porosity. At 4 wt.% Mo_2_C, the grain size further reduces to 0.1 μm–1.19 μm with more uniform binder distribution and increased volume fraction. The progressive grain refinement and enhanced binder volume fraction with increasing Mo_2_C content can be attributed to multiple mechanisms. First, the formation of (W,Mo)C solid solution through Mo_2_C dissolution into the WC lattice introduces lattice distortion that reduces the energy difference between grain interiors and boundaries, thereby diminishing the driving force for grain growth [23,24]. Second, the preferential dissolution of Mo_2_C over WC in the Co binder phase reduces the dissolution-precipitation tendency of W and C elements, further suppressing grain coarsening [25]. Additionally, Mo improves the interfacial wettability between binder and hard phases, promoting uniform binder distribution, mitigating stress concentration, and enhancing overall coating performance. However, at 6 wt.% Mo_2_C, the grain size increases to 0.1 μm–1.56 μm with significantly elevated binder volume fraction. This abnormal microstructural evolution likely results from excessive interfacial reactivity between Mo_2_C and the binder phase, which shifts the dynamic dissolution-precipitation equilibrium and induces non-steady-state dissolution of the hard phase, ultimately leading to binder phase accumulation and hard phase size heterogeneity [26]. Such excessive binder content deteriorates the coating’s mechanical properties, particularly reducing high-temperature hardness.

**Figure 8:**
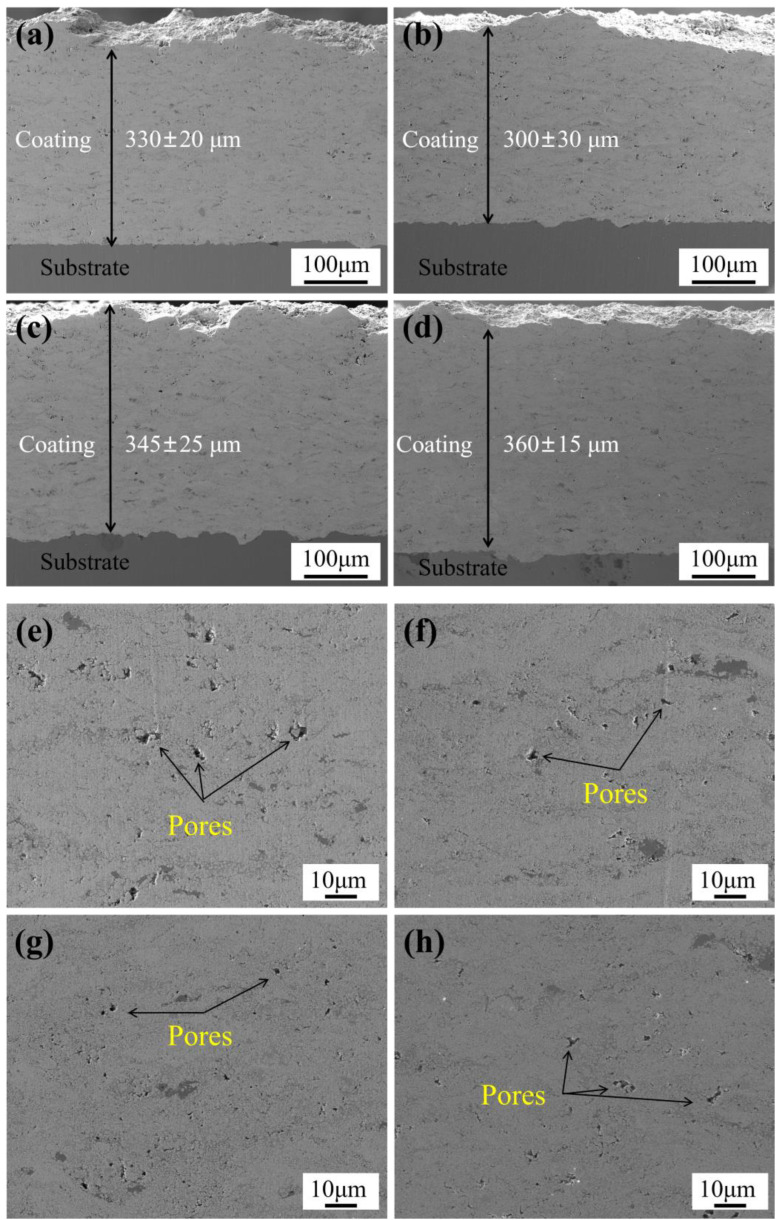
Cross-sectional SEM micrographs of coatings with different Mo_2_C additions: (**a**,**e**) 0 wt.% Mo_2_C; (**b**,**f**) 2 wt.% Mo_2_C; (**c**,**g**) 4 wt.% Mo_2_C; (**d**,**h**) 6 wt.% Mo_2_C.

**Figure 9:**
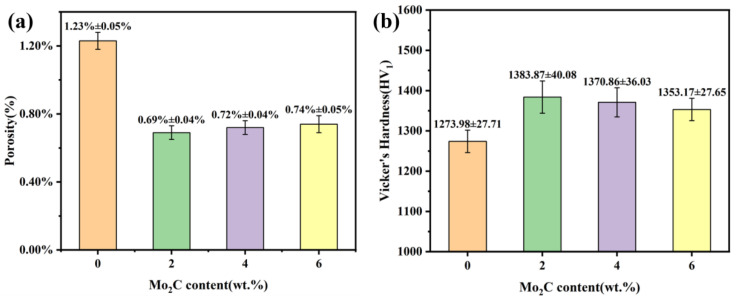
Porosity (**a**) and hardness (**b**) of WC-10Co4Cr coatings with different Mo_2_C additions.

Figure 8 shows the cross-sectional SEM micrographs of WC-10Co4Cr coatings with different Mo_2_C additions. As shown in Figure 8a–d, the coating thickness is approximately 300–360 μm. The coatings exhibit dense microstructures, uniform thickness, and smooth cross-sections without obvious cracks, confirming the appropriateness of the HVOF spraying parameters. The coatings bond well with the substrate, with no delamination or pores observed at the interface. The formation of a wavy line at the coating-substrate interface contributes to enhanced bonding strength. Figure 8e–h present higher-magnification SEM images of the coating cross-sections, revealing uniform microstructures where the dark regions represent pores. Comparative analysis indicates that the coating without Mo_2_C addition exhibits more pronounced porosity, with pore diameters measuring approximately 2–5 μm and a higher pore density. After Mo_2_C addition, the pore diameter decreases to about 1–3 μm with reduced pore count, while no significant differences are observed among coatings with varying Mo_2_C contents. Quantitative results in Figure 9a show that the coating without Mo_2_C addition has a porosity of 1.23 ± 0.05%, whereas coatings with 2 wt.%, 4 wt.%, and 6 wt.% Mo_2_C additions demonstrate porosity values of 0.69 ± 0.04%, 0.72 ± 0.04%, and 0.74 ± 0.05%, respectively. The addition of Mo_2_C leads to a notable decrease in coating porosity. When the Mo_2_C content increased from 2 wt.% to 6 wt.%, the change in porosity fell within the margin of error and was statistically insignificant, confirming that Mo_2_C contributes to porosity reduction and density improvement. This porosity decrease results from Mo enhancing the interfacial wettability between the binder and hard phases. The formed (W,Mo)C solid solution reduces liquid-solid interfacial tension, thereby directly improving wettability [27]. Through this enhanced wettability, the liquid phase can more completely fill interparticle voids among hard phase particles, thereby reducing pore formation, decreasing porosity, and improving coating density.

Figure 9b shows the hardness of WC-10Co4Cr coatings with different Mo_2_C additions. The addition of Mo_2_C increased the Vickers hardness of the coating, although a slight decrease in hardness was observed as the Mo_2_C content increased further. The maximum hardness of 1383.87 ± 40.08 HV is achieved at 2 wt.% Mo_2_C. The addition of Mo_2_C enhances coating hardness through multiple strengthening mechanisms. Primarily, Mo_2_C dissolves into the WC lattice to form a (W,Mo)C solid solution, inducing substantial lattice distortion. This distortion increases resistance to dislocation motion, impeding slip systems and thereby improving strength and hardness. Simultaneously, it suppresses the dissolution-reprecipitation process of WC grains, effectively inhibiting grain growth. The resulting grain refinement follows the Hall-Petch relationship, where increased grain boundary density strengthens resistance to dislocation movement, further contributing to hardness improvement [28,29]. Furthermore, Mo from Mo_2_C enhances coating density. The improved density increases the effective load-bearing cross-sectional area, consequently elevating coating hardness [30]. However, with excessive Mo_2_C addition, hardness begins to decline due to the corresponding increase in binder phase volume fraction and relative reduction in hard phase content. As the Mo_2_C content increases, the relative WC content in the coating decreases. Since both the added Mo_2_C and the formed (W,Mo)C solid solution possess lower hardness than WC, this contributes to an overall decrease in coating hardness. In summary, Mo_2_C addition enhances coating hardness through solid solution strengthening, grain refinement, and improved density, while the countervailing effect of increased binder phase fraction and decreased WC content at higher Mo_2_C contents leads to hardness reduction. The optimal balance is achieved at 2 wt.% Mo_2_C addition.

### 3.3. Wear Behavior of the HVOF Coatings

#### 3.3.1. At Room Temperature

**Figure 10:**
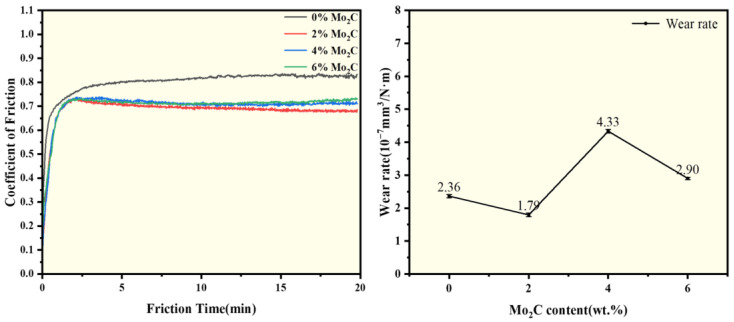
Friction-wear curves and wear rates of coatings with different Mo_2_C additions at room temperature.

**Figure 11:**
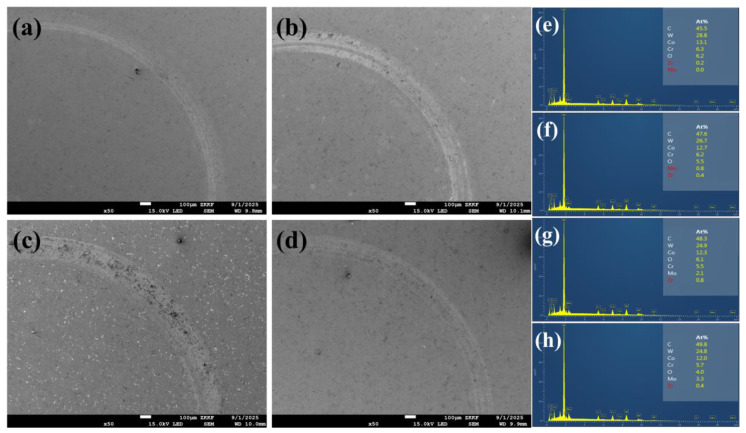
SEM micrographs and EDS spectra of wear tracks for WC-10Co4Cr coatings with different Mo_2_C additions at room temperature: (**a**,**e**) 0 wt.% Mo_2_C; (**b**,**f**) 2 wt.% Mo_2_C; (**c**,**g**) 4 wt.% Mo_2_C; (**d**,**h**) 6 wt.% Mo_2_C.

Figure 10 presents the friction-wear curves and wear rates of WC-10Co4Cr coatings with different Mo_2_C additions at room temperature. The friction-wear curves reflect the corresponding relationship between the friction coefficient and testing time during the wear process. All samples undergo two distinct stages: a running-in period and a stable period. During the running-in stage, contact between the grinding ball and coating surface generates substantial wear debris, causing a sharp increase in the friction coefficient. After a certain period, the wear debris stabilizes, and the friction coefficient subsequently levels off [31]. The coating without Mo_2_C addition exhibits a higher friction coefficient and longer running-in stage compared to those containing Mo_2_C. However, the friction coefficient increases with further increases in Mo_2_C content. The wear rate of the coatings exhibits a complex trend with increasing Mo_2_C content, initially decreasing, then increasing, and finally decreasing again. The minimum wear rate is achieved at 2 wt.% Mo_2_C addition. This optimal performance is attributed to the enhanced hardness and density imparted by Mo_2_C, which reduces wear debris generation, facilitates earlier transition to the stable friction stage, and results in a lower friction coefficient. Additionally, the formation of Mo-containing oxides during the wear process further contributes to reducing both friction coefficient and wear rate [32,33]. The wear resistance of the molybdenum element also contributes partly to the enhanced wear resistance of the coating. However, excessive Mo_2_C content leads to deterioration in coating hardness, consequently increasing both friction coefficient and wear rate. The anomalous increase in wear rate observed at 4 wt.% Mo_2_C may be attributed to the onset of adhesive wear mechanisms. Figure 11 presents the SEM images and EDS spectra of the wear tracks for WC-10Co4Cr coatings with different Mo_2_C additions at room temperature. The results indicate that the wear track widths of coatings with different compositions are generally similar, with more pronounced wear tracks observed in Figure 11b,c. EDS analysis of the wear tracks reveals the presence of Zr and O elements, suggesting intrusion of the grinding ball material into the coating surface during friction. The oxygen content exceeds the stoichiometric proportion in ZrO_2_, indicating that oxidation occurred during the friction process.

**Figure 12:**
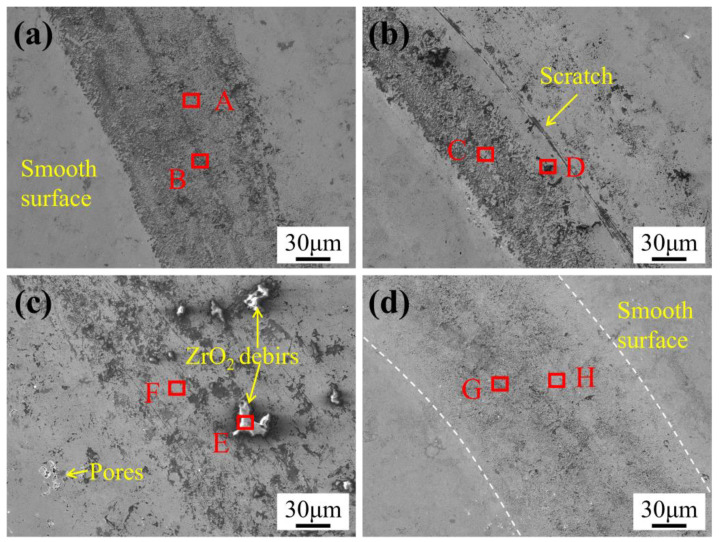
SEM micrographs of worn surfaces of WC-10Co4Cr coatings with different Mo_2_C additions at room temperature: (**a**) 0 wt.% Mo_2_C; (**b**) 2 wt.% Mo_2_C; (**c**) 4 wt.% Mo_2_C; (**d**) 6 wt.% Mo_2_C.

Figure 12 presents high-magnification SEM micrographs of the worn surfaces of WC-10Co4Cr coatings with different Mo_2_C additions at room temperature, with corresponding elemental compositions at selected points provided in Table 2. The wear tracks exhibit discontinuous dark regions where elemental analysis reveals significantly lower C and W concentrations compared to the gray areas. Combined with morphological characteristics, these dark regions are identified as cavities resulting from the pull-out of carbide particles, indicating that abrasive wear is the primary mechanism. Simultaneously, the elevated O and Zr contents within these cavities suggest the embedding of fine debris delaminated from the counter body during the wear process. The oxygen content at all analyzed points exceeds the stoichiometric ratio of ZrO_2_, demonstrating the concurrent occurrence of oxidative wear during sliding. As shown in Figure 12a, the coating without Mo_2_C addition displays the most extensive areas of carbide pull-out cavities, consistent with its relatively poor wear resistance. In contrast, the coating with 2 wt.% Mo_2_C addition (Figure 12b) shows significantly reduced dark regions and a lower density of spalling pits, along with notably narrower wear tracks. The observed scratches on the coating surface are likely caused by detached ZrO_2_ debris acting as third-body abrasives. This improvement is results from the enhanced hardness and density from Mo_2_C addition, which promotes a more uniform distribution of the hard phase within the binder phase. This effectively reduces carbide particle pull-out and mitigating abrasive wear, consequently leading to lower friction coefficient and wear rate. With further increase in Mo_2_C content, the wear track width expands. Figure 12c reveals distinct bright areas identified as adhered ZrO_2_ debris through elemental analysis. This suggests that the improved coating hardness and density enable the material to tear fragments from the counter surface during friction, thereby inhibiting further penetration of the grinding ball and slowing the wear process. However, these captured ZrO_2_ debris adhere to the wear track under combined frictional pressure and temperature, subsequently undergoing material transfer and tearing under shear stresses, which induces adhesive wear and increases both friction coefficient and wear rate. The microcracks forming around the ZrO_2_ debris serve as precursors to material spalling and delamination. Although the wear track in Figure 12d appears less distinct, its width remains considerable with fine spalling pits, accompanied by increased friction coefficient and wear rate. This phenomenon results from the abnormal increase in binder phase volume fraction and the partial dissolution-refinement of the hard phase, leading to more severe material removal while generating finer spalling pits that make the wear track less distinct.

#### 3.3.2. At 400 °C

**Figure 13:**
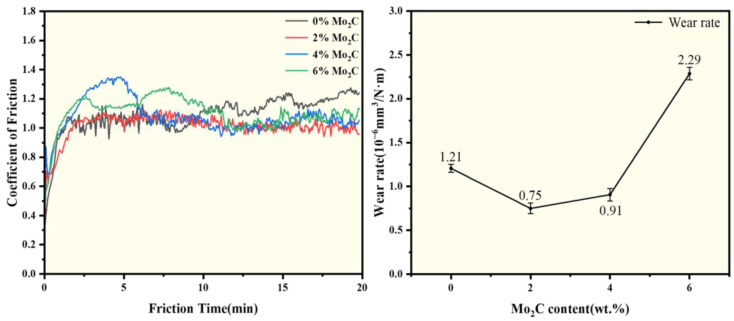
Friction-wear curves and wear rates of coatings with different Mo_2_C additions at 400 °C.

**Figure 14:**
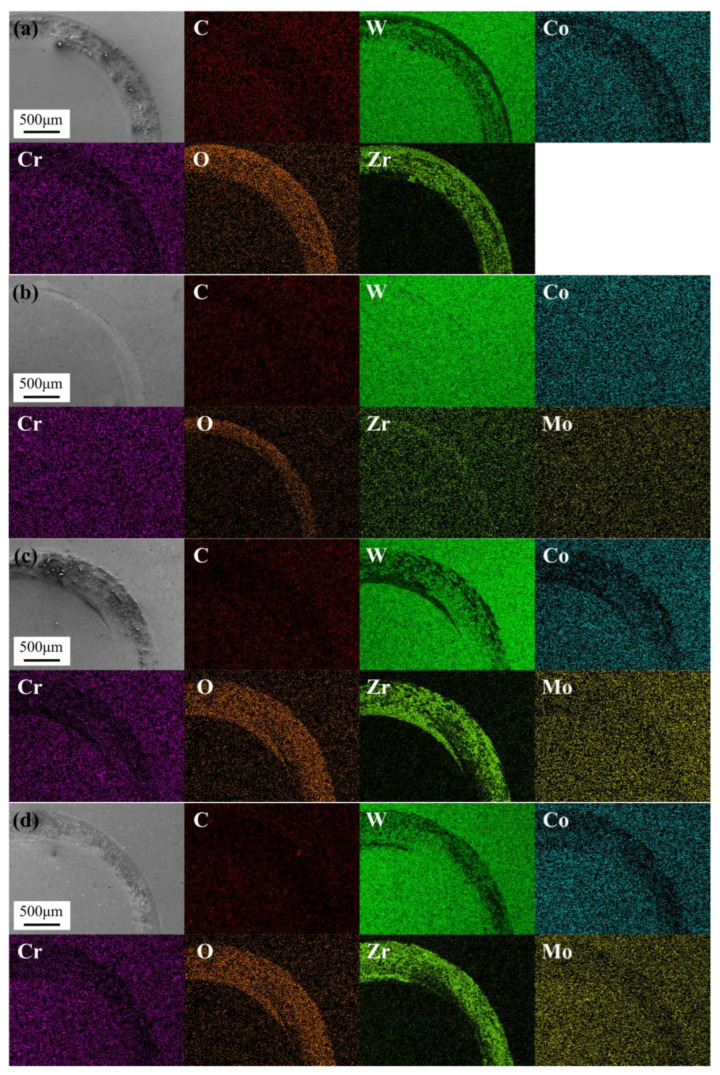
SEM micrographs and elemental distribution maps of wear tracks for WC-10Co4Cr coatings with different Mo_2_C additions at 400 °C: (**a**) 0 wt.% Mo_2_C; (**b**) 2 wt.% Mo_2_C; (**c**) 4 wt.% Mo_2_C; (**d**) 6 wt.% Mo_2_C.

Figure 13 presents the friction-wear curves and wear rates of WC-10Co4Cr coatings with different Mo_2_C additions at 400 °C. Compared with Figure 10, the friction coefficient curves obtained at 400 °C exhibit more pronounced fluctuations and higher average values. This performance degradation is attributed to the thermal softening of the metallic binder phase at elevated temperatures, which reduces the overall material hardness, increases the contact area with the counterface, and exacerbates plastic deformation and material removal, consequently elevating the friction coefficient [34,35]. At 400 °C, the coating with 2 wt.% Mo_2_C addition demonstrates the lowest friction coefficient and wear rate. The wear rate initially decreases then increases with rising Mo_2_C content, corresponding well with the variations in coating hardness and density. Figure 14 shows the SEM morphologies and elemental distribution maps of the wear tracks for WC-10Co4Cr coatings with different Mo_2_C additions at 400 °C. Compared with Figure 11, the wear tracks at 400 °C are wider and exhibit more severe damage. The coating with 2 wt.% Mo_2_C addition displays narrower wear tracks and less severe damage compared to other coatings. Elemental analysis of the wear tracks shows elevated oxygen content, slightly increased zirconium levels, and minor reductions in W and C elements, indicating only minimal pull-out of WC hard phase with oxidation as the dominant process. EDS analysis of other coatings reveals decreased concentrations of C, W, Co, Cr, and Mo alongside increased O and Zr content in the wear tracks, confirming that these coatings experienced significant binder phase loss and WC particle extraction during wear, accompanied by pronounced oxidative wear. Based on the varying trends in friction coefficients, wear rates, and elemental distribution characteristics across different coatings, it is concluded that three distinct wear mechanisms operate during the high-temperature wear process.

**Figure 15:**
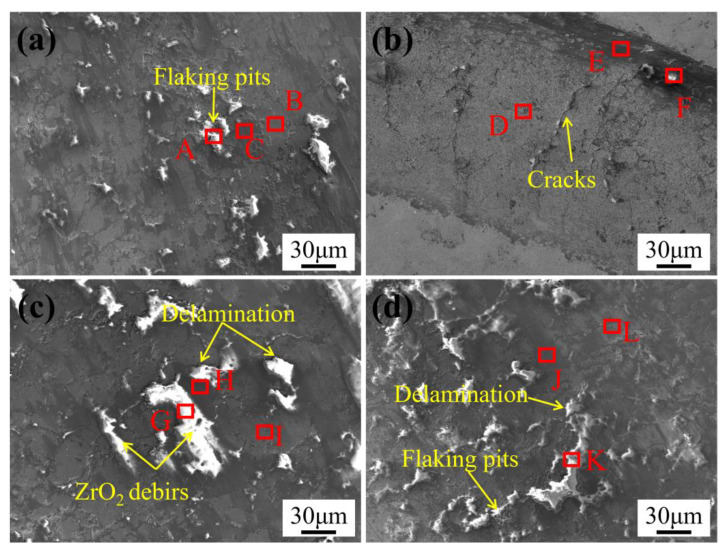
SEM micrographs of worn surfaces of WC-10Co4Cr coatings with different Mo_2_C additions at 400 °C: (**a**) 0 wt.% Mo_2_C; (**b**) 2 wt.% Mo_2_C; (**c**) 4 wt.% Mo_2_C; (**d**) 6 wt.% Mo_2_C.

Figure 15 presents high-magnification SEM micrographs of the worn surfaces of WC-10Co4Cr coatings with different Mo_2_C additions tested at 400 °C, while Table 3 provides the corresponding elemental composition analysis that elucidates wear mechanism transitions. As shown in Figure 15a, the coating without Mo_2_C addition displays a characteristic flaky morphology on its worn surface, consisting of bright white and dark gray regions embedded within a light gray matrix. Elemental analysis reveals that the bright white regions are rich in O and Zr, the dark gray regions contain elevated concentrations of O, Zr, and W, while the light gray matrix is predominantly composed of W. Based on these morphological and elemental characteristics, the bright white regions are identified as adhered ZrO_2_ wear debris. During prolonged sliding, these debris particles combine with abrasive grains to form the dark gray transitional regions, which eventually spall off to expose the underlying light gray matrix. The observed micropores within this matrix originate from pulled-out WC particles, confirming that abrasive wear and adhesive wear coexist as primary mechanisms. In contrast, the coating with 2 wt.% Mo_2_C addition (Figure 15b) exhibits a significantly improved worn surface morphology. The worn surface is primarily composed of light gray regions with narrow dark gray zones along the wear track edges. Although no distinct flaky structures are observed, distinct microcracks are visible. Elemental mapping shows relatively high W and O concentrations in these areas, indicating minimal large-scale WC particle spalling and effective suppression of abrasive wear. The uniform oxygen distribution throughout the wear track further demonstrates that oxidative wear has become the dominant mechanism. These microcracks likely result from the thermal softening of the binder phase at elevated temperatures and the associated compressive stresses. With 4 wt.% Mo_2_C addition (Figure 15c), the worn surface comprises bright white, dark gray, and light gray regions, where the bright white and dark gray areas expand significantly compared to the coating without Mo_2_C, accompanied by evident delamination. The composite layer formed by ZrO_2_ debris and abrasive grains remains largely intact without massive spallation. This phenomenon is attributed to the lubricating effect of the in-situ generated Mo-containing oxides (likely predominantly in the form of MoO_3_) during friction, which effectively reduces interfacial shear stress and suppresses adhesive wear [36]. Notably, the coating with 6 wt.% Mo_2_C addition (Figure 15d) shows increased bright white regions along with numerous spalling pits and significantly enhanced surface roughness, indicating severe adhesive wear. This deterioration directly correlates with the abnormally increased binder phase volume fraction and the consequent hardness reduction. However, the continuous formation of the Mo-containing oxides eventually inhibits the adhesive wear process, consistent with the descending trend observed in the friction coefficient curve.

#### 3.3.3. Wear Mechanism

**Figure 16:**
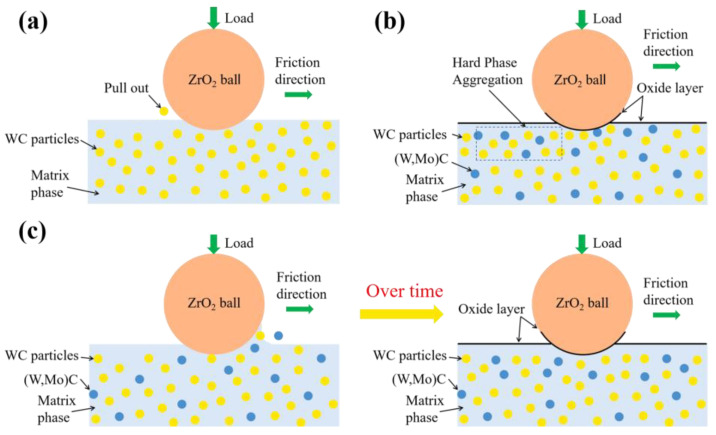
Schematic diagram of wear mechanisms for different coatings at 400 °C: (**a**) 0 wt.% Mo_2_C; (**b**) 2 wt.% Mo_2_C; (**c**) 4 wt.% Mo_2_C and 6 wt.% Mo_2_C.

Systematic examination of worn surfaces reveals three distinct wear mechanisms in WC-10Co4Cr coatings at 400 °C, with their transition closely dependent on Mo_2_C content, as schematically illustrated in Figure 16. The coating without Mo_2_C addition (Figure 16a) demonstrates the most complex wear behavior. Under high-temperature conditions, initial abrasive wear causes massive WC particle detachment, forming spalling pits. As sliding progresses, the edges of these pits initiate severe adhesive wear while simultaneous surface oxidation and oxide layer delamination occur. The liberated WC particles and oxide debris act as third-body abrasives at the friction interface, creating a three-body wear effect. This synergistic interaction of multiple wear mechanisms results in progressively increasing friction coefficients beyond the running-in period. With 2 wt.% Mo_2_C addition (Figure 16b), the wear mechanism undergoes a significant transformation. The enhanced coating hardness and density effectively suppress abrasive wear, minimizing WC particle detachment. At elevated temperatures, WC particles become embedded within the binder phase, resisting grinding ball penetration [37]. Concurrently, the in-situ formed Mo-containing oxides (likely predominantly in the form of MoO_3_) provide lubrication, yielding the lowest and most stable friction coefficient with a gradual declining trend. The uniform oxygen distribution throughout wear tracks confirms oxidative wear as the dominant mechanism. At higher Mo_2_C contents (Figure 16c), new wear characteristics emerge. When the Mo_2_C addition is excessive, the hardness of the coating exhibits a slight declining trend, concurrently with a corresponding reduction in wear resistance. When Mo_2_C is added in an appropriate amount, it dissolves into the WC lattice to form a (W,Mo)C solid solution. This is accompanied by solid-solution strengthening and grain-refinement strengthening effects, which play a key role in enhancing the coating hardness. However, when the Mo_2_C content continues to increase to an excessive level, the dynamic dissolution-precipitation equilibrium of the system shifts. This leads to non-steady-state dissolution of the hard phase, abnormal growth in the volume fraction of the binder phase, and a corresponding reduction in the volume proportion of the hard phase. Such structural changes weaken the coating’s ability to resist abrasive indentation and plastic cutting, resulting in a reversion of the wear mechanism to adhesive wear. During initial wear, the grinding ball penetrates deeper into the coating surface, causing immediate material delamination and consequently higher initial friction coefficients. As wear progresses, generated debris adheres to the wear track under combined thermal and pressure effects, forming protective layers that inhibit further damage. This stage involves noticeable adhesive wear with numerous spalling pits. With continued Mo-containing oxides formation, the friction coefficient gradually decreases and stabilizes, transitioning to a hybrid mechanism dominated by oxidative wear with supplementary adhesive wear, resulting in wider wear tracks and heterogeneous elemental distribution.

The wear behavior of WC-10Co4Cr coatings at 400 °C primarily involves competitive and synergistic interactions among abrasive, oxidative, and adhesive wear mechanisms. Mo_2_C additions enhance coating hardness and density through solid-solution strengthening and grain refinement, effectively suppressing abrasive wear. Simultaneously, the promoted Mo-containing oxides function as a solid lubricant that not only reduces friction coefficients but also isolates contacting surfaces, thereby mitigating both oxidative and adhesive wear. However, excessive Mo_2_C content induces abnormal binder phase accumulation and hardness reduction, shifting wear mechanisms toward adhesive wear dominance and exhibiting complex nonlinear evolution patterns. These findings provide crucial theoretical guidance for designing advanced high-temperature wear-resistant coatings.

## 4. Conclusions

This study employed high-velocity oxygen-fuel (HVOF) spraying to deposit WC-10Co4Cr coatings with different Mo_2_C additions onto 316L stainless steel substrates, investigating the influence of Mo_2_C content on the microstructure and tribological properties of the coatings. The main conclusions are as follows:

(1) The addition of Mo_2_C to the feedstock powder did not alter the powder microstructure. The powders exhibit spherical morphology with good flowability and apparent density, and possess a particle size of approximately 15–45 μm. The powder is primarily composed of the WC phase, along with minor amounts of Co, Cr and W_2_Co_4_C phases. After Mo_2_C addition, the diffraction peak intensities of WC and Co decrease, indicating the formation of a solid solution.

(2) The coatings have no significant defects and exhibit a dense microstructure without cracks or delamination. The coatings are primarily composed of WC hard phase and CoCr binder phase. With Mo_2_C addition, a solid solution forms, the grain size of the WC particles is refined, and the distribution of the binder phase becomes more uniform. When the Mo_2_C content reaches 2 wt.%, the coating achieves the lowest porosity of 0.69 ± 0.04%. With a further increase in Mo_2_C content, the porosity shows a slight rise. Similarly, the highest average microhardness of 1383.87 ± 40.08 HV is obtained at 2 wt.% Mo_2_C addition, beyond which the hardness decreases. The addition of Mo_2_C enhances coating hardness and reduces porosity.

(3) Proper Mo_2_C addition improves the room temperature wear resistance of the carbide-metal coating. At room temperature, the friction coefficient of the coatings first decreased and then increased with increasing Mo_2_C content, while the wear rate initially decreased, then increased, and finally decreased again. The coating with 2 wt.% Mo_2_C exhibited the lowest friction coefficient, approximately 0.68. The primary wear mechanism of the coating is abrasive wear. The addition of Mo_2_C enhances the hardness and density of the coating, thereby mitigating abrasive wear and improving wear resistance. However, it also induces adhesive wear, which becomes more severe with increasing Mo_2_C content.

(4) Proper Mo_2_C addition improves the 400 °C wear resistance of the carbide-metal coating. Experimental results demonstrate that both the friction coefficient and wear rate exhibit a characteristic initial decrease followed by an increase with rising Mo_2_C content. The most favorable tribological performance, characterized by the lowest and most stable friction coefficient, is achieved at 2 wt.% Mo_2_C. This performance optimization correlates directly with fundamental transitions in wear mechanisms. The coating without Mo_2_C addition displays complex wear behavior involving simultaneous abrasive, adhesive, and oxidative wear. At the optimal 2 wt.% Mo_2_C content, the wear mechanism undergoes a significant transition to oxidative wear dominance. This transition is facilitated by enhanced coating hardness and density, thereby suppressing abrasive wear, while the in-situ-generated Mo-containing oxides act as a solid lubricant. However, excessive Mo_2_C addition introduces detrimental effects. The abnormal increase in binder phase volume fraction and consequent hardness reduction lead to significantly higher initial friction coefficients and wear rates, where adhesive wear becomes the primary mechanism. As the wear process advances, the continuous formation of Mo-containing oxides moderates this effect, establishing a hybrid wear mechanism dominated by oxidative wear with supplementary adhesive wear. This mechanistic understanding provides crucial insights for designing high-performance coatings for elevated temperature applications.

## Figures and Tables

**Table 1:** Nominal composition of the Feedstock powder.

Sample Number	WC (wt.%)	Co (wt.%)	Cr (wt.%)	Mo_2_C (wt.%)
C1	Bal	10	4	0
C2	Bal	10	4	2
C3	Bal	10	4	4
C4	Bal	10	4	6

**Table 2:** EDS analysis of worn surfaces at room temperature.

Points	Elements (wt.%)						
	C	W	Co	Cr	O	Zr	Mo
A	8.67	73.95	10.19	2.02	4.29	0.88	0
B	4.78	56.53	7.81	20.67	8.11	2.10	0
C	6.72	70.87	11.50	1.50	6.35	3.06	0
D	4.49	57.66	7.83	5.53	11.20	12.45	0.85
E	6.30	33.69	6.15	2.51	21.79	28.10	1.47
F	9.18	76.73	9.31	1.24	1.72	0.82	1.00
G	7.28	64.89	9.24	4.63	8.24	1.95	3.76
H	5.13	73.17	9.77	2.59	2.14	0.47	6.73

**Table 3:** EDS analysis of worn surfaces at 400 °C.

Points	Elements (wt.%)						
	C	W	Co	Cr	O	Zr	Mo
A	4.74	13.26	4.13	1.25	11.7	64.91	0
B	13.25	66.80	16.17	1.08	2.00	0.69	0
C	5.01	37.04	1.94	0.64	16.23	40.14	0
D	4.60	54.81	21.73	5.07	9.92	2.31	1.56
E	3.90	58.59	8.22	3.31	16.67	8.36	0.96
F	5.89	50.38	9.37	4.07	18.87	9.87	1.55
G	7.40	10.43	2.55	0.98	21.44	57.19	0
H	5.79	24.86	3.86	0.91	15.01	49.15	0.42
I	6.14	69.18	7.48	1.99	6.07	7.68	1.47
J	6.01	34.53	4.89	3.07	15.34	34.07	2.10
K	7.87	13.44	3.03	1.13	21.18	53.10	0.26
L	5.13	52.70	4.04	2.25	11.42	18.58	5.88

## Data Availability

The original contributions presented in this study are included in the article. Further inquiries can be directed to the corresponding authors.
